# Synthetic *Manihot esculenta* Rubisco activase proteins with increased thermotolerance identified via machine learning

**DOI:** 10.17912/micropub.biology.001773

**Published:** 2025-10-24

**Authors:** Clayton Dilks, Rhiannon LaVine, Claire Buchanan, Daniel Russo, Elizabete Carmo-Silva

**Affiliations:** 1 Evozyne Inc, Chicago, Illinois, United States; 2 Environment Centre, Lancaster University, Lancaster, England, United Kingdom

## Abstract

Adaptation to increasing environmental temperatures is essential to plant survival and human food production. Thermal tolerance is controlled by a complex network of factors in plants including but not limited to genetic variation and environmental context. Rubisco activase (Rca) is a key photosynthetic enzyme with low thermal tolerance. Here, we report a large machine learning-directed screen of >1,400 synthetic cassava Rca enzymes which identified mutations that convey increased thermal stability while minimizing introduced mutations. We demonstrate multiple synthetic proteins that maintain activity at 8°C higher than wildtype cassava Rca including a single mutation that retains most activity post heat-shock.

**Figure 1. Thermotolerant synthetic Rubisco activase (Rca) proteins with minimal mutations identified via a large machine learning-directed screen.**
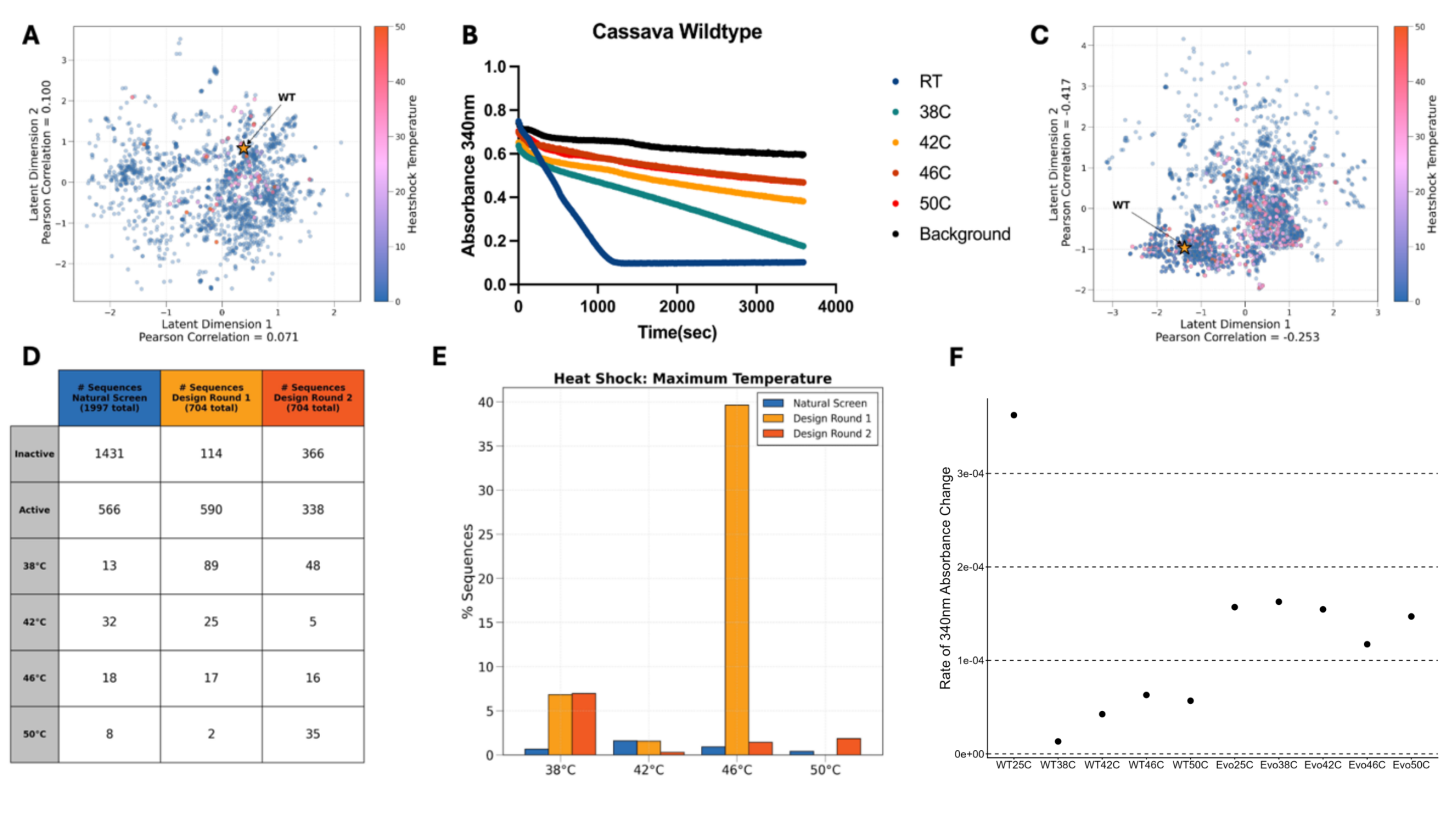
(A) Latent space showing two out of four latent dimensions with each Rca sequence color indicative of the highest thermal challenge under which it maintained ATPase activity. The wildtype cassava (WT) is indicated with a gold star. This latent space has not been trained on any data so low correlation is observed. (B) Wildtype Cassava Rca activity at the temperature indicated in the legend (the 46°C and 50°C traces are overlapping). The decrease in absorbance at 340 nm is proportional to the rate of ATP hydrolysis by Rca. (C) Latent space after training using Variational Autoencoder (VAE) deep generative models again showing two of the four latent dimensions each point representing a sequence and the color indicating the maximum temperature at which residual activity was observed. (D) The number of Rca sequences tested in each screening/design round that were active or inactive at room temperature, and that maintained activity at each heat shock temperature. (E) The percentage of sequences which maintained activity up to each heat shock temperature across each screening/design round. (F) The ATP hydrolysis activity of our top performing sequence compared to the wildtype cassava Rca sequence (Rca) with the heat shock temperature and sequence indicated on the x-axis.

## Description

Food insecurity is a major problem for millions of people globally. Recent estimates suggest that over 800 million people do not have a secure supply of food (Qu et al., 2023). As climate change is predicted to decrease the production of many essential crops around the world, food insecurity is expected to grow. These crops include wheat (Qu et al., 2023), maize (Li et al., 2022), rice (Saud et al., 2022), cassava (Pipitpukdee et al., 2020), and many others. Cassava is an essential staple in the diet of millions of people in equatorial regions (Tize et al., 2021). Cassava grows optimally between 25-29°C and, although it can withstand temperatures up to 40°C, the yield is greatly reduced.

A promising avenue for crops is the engineering of Rubisco and its associated chaperones. Rubisco is one of the most abundant proteins in the world and is essential for conversion of atmospheric CO _2_ into energy within plants, algae and cyanobacteria. A known limitation of Rubisco is the propensity for the complex to be inhibited by the incorrect binding of sugar phosphates (Mueller-Cajar, 2017). As temperatures rise, the concentration of inhibitory molecules also increases, which causes an overall decrease in photosynthetic rate (Waheeda et al., 2023). Rubisco activase (Rca), a chaperone of Rubisco, functions by hydrolyzing ATP and tying the energy released to the removal of the inhibitory molecules from Rubisco catalytic sites (Waheeda et al., 2023). Previous research has shown that the introduction of a more thermostable Rca increases the plant’s ability to withstand higher temperatures (Kurek et al., 2007; Kumar et al., 2009). We aim to increase the thermal tolerance of the *Manihot esculenta* (cassava) Rca with three rounds of machine learning-directed engineering paired with a high-throughput assay which measures ATPase activity after a thermal challenge.

There are four Rca genes (XP_021625935.1, XP_021625936.1, XP_021624073.1, and XP_021628250.1) encoded in the cassava genome. XP_021625935.1 and XP_021625936.1 are splice variants that are identical besides a C-terminal extension involved in redox regulation. XP_021628250.1 encodes a more divergent and shorter protein than the other three Rca proteins. XP_021624073.1 is the gene we chose to focus our engineering efforts on because it does not have splice variants to consider, and it readily expressed in *E. coli* compared to the other cassava Rcas. All these genes were used as query sequences to retrieve orthologs to populate a multiple sequence alignment (MSA). The PSIBLAST query yielded 3,915 unique sequences (Schäffer et al., 2001; Altschul et al., 1997). 245,710 additional unique sequences were retrieved from the Mgnify database (Richardson et al., 2023) to supplement the dataset. Next, the dataset was refined to contain only sequences with greater than 20% identity to a reference sequence. This filter reduced the set to 2,573 sequences with a minimum length of 140 amino acids. Denmark Technical University’s TargetP 2.0 model (Armenteros et al., 2019) was used to predict chloroplast transit peptides (cTP) in the sequences. 1,852 sequences were identified to contain a cTP which was removed prior to the preliminary alignment with FAMSA (Deorowicz et al., 2016). Further quality assurance and trimming steps were performed on the sequences (details in methods) before a final set of 1,997 sequences was chosen for initial temperature screening, referred to herein as the “natural screen” round.

Variational Autoencoders (VAEs) are deep generative models composed of an encoder and a decoder. The encoder compresses complex protein sequence data into a low-dimensional latent space, which the decoder then uses to reconstruct the input sequences. During training, VAEs use variational inference to optimize a loss function that balances reconstruction accuracy and regularizes the latent space with a prior distribution (Kingma and Welling, 2013, 2019; Zhao et al., 2019). An additional semi-supervised prediction layer can be included to predict experimentally measured targets, which enables the model to be iteratively re-trained with new data from synthesized sequences (Castro et al., 2018; Gómez-Bombarelli et al., 2018; Frassek et al., 2021; Lian et al., 2022). This approach allows the latent space to capture key patterns of amino acid mutations that characterize the sequence set of interest and provide an interpretable, low-dimensional embedding to facilitate design of novel sequences with desirable functionality (Sevgen et al., 2023). An unsupervised VAE was trained on the MSA, resulting in no strong latent space organization with respect to thermal tolerance of the Rca sequences ( Figure 1A ). Gaussian sampling was performed to generate synthetic Rca variants within 20 hamming distance of the primary wildtype (XP_021624073.1) to further characterize the sequence of interest and provide training data for design round 1.

The respective DNA sequences were obtained from IDT, cloned, expressed recombinantly, and the proteins were purified in high throughput (see methods). After purification, we used an ATPase activity assay that couples the production of ADP to the oxidation of NADH to NAD+ which can be monitored through the decrease in absorbance at 340 nm (Barta et al., 2011). At 5 µM the wildtype Rca sequence demonstrated robust activity at room temperature (RT) but lost activity when subject to a heat shock at 42°C or higher temperatures ( Figure 1B (the 46°C and 50°C traces are overlapping)). We found 566 sequences from the natural screen that showed ATPase activity at room temperature ( Figure 1C /D). We also performed a series of temperature challenges to identify the thermal tolerance of each Rca sequence tested. For these tests, we placed 20 µL of purified protein into a 384-well plate and placed the plate into a pre-equilibrated thermocycler (38°C, 42°C, 46°C, and 50°C) for one hour. After temperature challenges, the samples were centrifuged (2,000 rcf for 2 min) and then 10 µL of sample was moved into a plate pre-loaded with 40 µL of assay buffer and absorbance was measured after ~16 hours in assay buffer. This permissive screen was performed to give sequences with low activity the opportunity to complete the reaction if any active protein remained post heat shock. We found that 92 sequences remained active after any of these temperatures, 18 of which were active above 46°C. Sequential design rounds (Design Round 1 and Design Round 2) proceeded with training a semi-supervised VAE using ATPase activity data. The resultant latent spaces showed more thermotolerance-related organization ( Figure 1C ), allowing for data-informed generation of synthetic Rca sequences with the desired thermal tolerant phenotype.

After the model and sequence generation were completed, these sequences were tested for activity as described above with one additional screening step. These sequences were concentration-normalized prior to thermal challenges. We first measured the concentration with the high-throughput microfluidic system (Revvity LabChip). After the concentration was measured, the samples were diluted in elution buffer to 5 µM of protein in 6 µL total with the ECHO liquid handling system. Sequences with a concentration <5 µM were run at the highest possible concentration. This set of sequences showed an 83.8% active rate (590/704 tested) and showed activity even at the highest tested temperatures. We identified 19 sequences with activity after a 46°C temperature challenge, two of which were active after 50°C. These data were included in the semi-supervised VAE and a second design round was generated.

The sequences generated in design round two were screened in the same manner as design round one. We found 35 additional sequences that had activity after a 50°C heat shock, some of which retained close to 100% activity after the challenge at the highest temperature ( Figure 1D /E/F). A sequence of particular interest is designated as evozyne_rca-1 (EVO in Figure 1F ), this sequence has only a single mutation from the wildtype sequence with a proline to glycine swap at position 250 after transit peptide removal. A notable observation with this sequence is that increasing thermal stability has led to a loss in overall activity, a trade off which has been previously documented ( Figure 1F ) (Degen et al., 2020; Vanella et al., 2024). The remaining sequences from design rounds 1 and 2 as well as the natural round are included in Data table 1, along with if each sequence showed activity and the highest temperature at which the protein retained activity.

One difficulty of engineering enzymes with increased thermostability is that we currently do not know the level of Rca activity needed *in planta* . If only a small amount of activity is enough to cause a large improvement in crop stability, this increase may be enough to improve crop yield. If activity equivalent to the wildtype Rca enzyme is required, additional engineering on the thermally stable sequences should be performed to maintain the stability gains while reintroducing the level of wildtype activity. Overall, we believe the engineered sequences presented here represent an excellent starting point for further engineering of cassava to maintain high yields in the face of climate change.

## Methods

**Reagents**

All chemicals including adenosine 5’ triphosphate (ATP), magnesium chloride (MgCl2), phosphoenyol-pyruvate (PEP), potassium chloride (KCL), polyethylene glycol (PEG), pyruvate-kinase (PK), lactate dehydrogense (LDH) lysozyme, DNAse, β-nicotinamide adenine dinucleotide reduced disodium salt (NADH), and imidazole were purchased from Sigma Aldrich. Other reagents were purchased from GoldBio or Thermo Fisher. Cloning and transformation reagents are purchased mainly from New England BioLabs and Teknova.

**Cloning**

A proprietary codon optimization algorithm was used to optimize the nucleic acid codon usage of the sequences for expression in *E. coli* BL21 (DE3) cells. After optimization, 5’ and 3’ adapters were added to each sequence which included cloning sites for introduction into a modified pDV4 plasmid using golden-gate cloning with BsaI. This cloning design put sequences in frame with a 6x HIS tag for purification and a HRV 3C protease site for tag removal. The sequences were then cloned into the modified pDV4 plasmid and transformed into BL21 (DE3) *E. coli.* The chaperone plasmid pGro7, which encodes the groEL and groES chaperones under an arabinose inducible plasmid, was used to assist in protein folding.

**Protein Expression**

Protein expression was initiated by inoculating the transformed cells in autoinduction media with selective antibiotics (6.7 g/L NaHPO _4_ , 3.4 g/L KH _2_ PO _4_ , 20 g/L tryptone, 5 g/L yeast extract, 5 g/L NaCl, 5 g/L dextrose, 20 g/L lactose, 6% w/v of 100% glycerol, 0.5 mg/mL arabinose, 100µg/mL carbenicillin, and 6.25µg/mL chloramphenicol) (Grabski & Drott, 2005). Cultures were grown at 37°C with shaking at 800RPM in 96-deep well plates until the optical density (OD600) of the culture reached ~0.8. The temperature was then dropped to 20°C with continuous shaking overnight for ~16 hours. After expression the plates were centrifuged at 4000 rcf for 10 minutes at 4°C, decanted, and then frozen at –80°C. Plates were thawed on ice and the cells were lysed using a SoluLyse-based system (0.10 µL/mL Lysonase, 5 mM DTT, 1 mM PMSF, 5 mM MgCl _2_ , 300 mM NaCl, 2 mM ATP) with manual pellet resuspension. The samples were clarified by centrifugation (60 minutes at 4°C, 5500 rcf).

**Ni-NTA Protein Purification**

Cell pellets were gently re-suspended in 120 μL of lysis buffer (50mM sodium phosphate, 90mM NaCl, 0.15mg/mL DNAse, 0.8mg/mL Lysozyme, 10mM MgCl2, 1mM PMSF, 2mM ATP, pH 7.8), transferred to 96-well PCR plates and held on ice for 10 minutes. PCR plates were then centrifuged at 5500 rcf, 4°C, for 60 minutes to pellet cellular debris in the sample. The clarified lysate was transferred to a deep well plate pre-loaded with 25uL of charged Ni-NTA magnetic beads (Genscript L00295) using the VIAFLOW liquid handler. Plates were then placed in a 4°C incubator, 800RPM for 60 minutes to bind the protein to the charged Ni-NTA beads. After this binding step, the plate is placed on a magnetic plate to allow removal of clarified lysate from the beads. To each well, 500 μL of wash buffer (1X PBS pH 7.8, 200mM NaCl, 50mM Imidazole, 2mM ATP, 3mM MgCl2) was added to wash the beads with an 800RPM, 4°C, 10 minutes incubation. The wash buffer is aspirated out by placing the deep well plate on the magna-rack. This wash step was repeated twice. The elution step consisted of 45 μL of elution buffer (1X PBS pH 7.8, 200mM NaCl, 50mM Imidazole, 2mM ATP, 3mM MgCl2) with an incubation time of 10 minutes, 4°C. Once the elution step is completed, the plate is placed again on the magna-rack and the eluted protein solutions transferred into a fresh 96-well PCR plate.

**ATPase activity assay**

For testing Rca activity using the ATPase assay, a reaction mixture of 45 μL containing 100mM Tris-KOH, 10mM MgCl _2_ , 20mM KCL, 5mM DTT, 2mM PEP, 5% w/v PEG, 300uM NADH, 5U/mL PK, 5.75U/mL LDH, and 2mM ATP is loaded into a 384-well optical plate using the VIAFLOW liquid handler (Barta et al., 2011). 5 μL of each eluted protein was added to the respective wells on the optical plate and mixed briefly with the liquid handler. The plate was quickly spun on a tabletop centrifuge for 30 seconds and read on a plate reader at 340 nm. The reaction couples ATP to ADP conversion to NADH to NAD+ oxidation using pyruvate kinase and lactate dehydrogenase. The NADH oxidation rate is continuously monitored by measuring the absorbance at 340 nm.

## Data Availability

Description: Dataset containing sequence IDs, sequences, information on each sequence, and activity data used throughout manuscript. . Resource Type: Dataset. DOI: https://doi.org/10.22002/kpejh-m5258
